# Supplementary material for: Universal and tunable liquid–liquid separation by nanoparticle-embedded gating membranes based on a self-defined interfacial parameter
Source: Nat Commun. 2021 Jan 4;12:80. doi: 10.1038/s41467-020-20369-9 (PMC7782719; doi:10.1038/s41467-020-20369-9)
Supplement: Supplementary file 1 — Supplementary Information [file 41467_2020_20369_MOESM1_ESM.pdf]

Supplementary Information

Universal and tunable liquid-liquid separation by nanoparticle-embedded gating membranes based on a new-defined interfacial parameter

Li et al.

Supplementary Figures

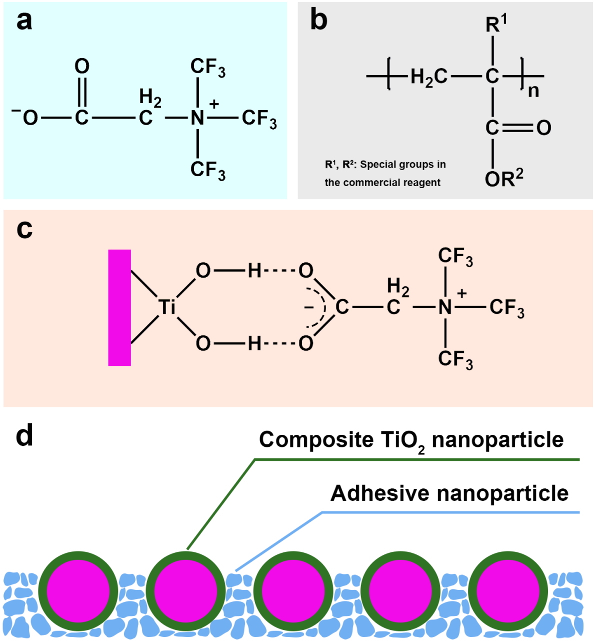

Supplementary Figure 1. The chemical structure of the used reagents and the schematic illustration of the self-assembly and adhesive mechanism. **a,** Capstone FS-50 is one kind of commercial amphoteric fluorosurfactant that the main chemical structure is derived from betaine. The corresponding chemical structure formula is inferred from the product description and the characterization methods in this work. **b,** The main active chemical composition of the used adhesive is crosslinking polyacrylate that is grafted by special groups as described in the product description and the relevant literature.[1] **c,** The hydrogen bond self-assembly process is performed between the surface of the TiO_2 nanoparticle and the fluorosurfactant molecule. **d,** The coating works in a synergistic way based on the two components. Mechanical interlocking occurs as the crosslinking adhesive nanoparticles flow into the pores on the SSM and the gaps between the composite TiO_2 nanoparticles. On the one hand, the interlocking improves the strength and durability of the as-prepared coating. On the other hand, it also serves to increase the total contact surface area between different nanoparticles to form the multi-re-entrant hierarchical structure, which is beneficial for enhancing the surface wettability of the coated membrane in Cassie-Baxter theory.

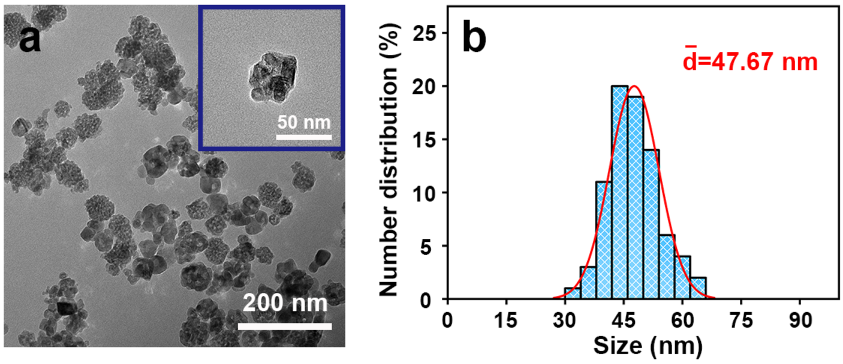

Supplementary Figure 2. TEM images of the composite TiO_2 nanoparticles with an average particle size of about 50 nm. **a,** Low-magnification and high-magnification TEM images of the measured nanoparticles. **b,** The size distribution and the average statistical size of the measured nanoparticles.

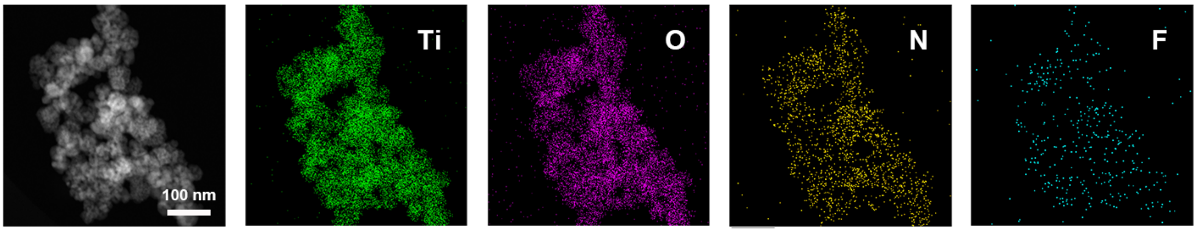

Supplementary Figure 3. High-resolution TEM images and elemental mapping of the composite TiO_2 nanoparticles.

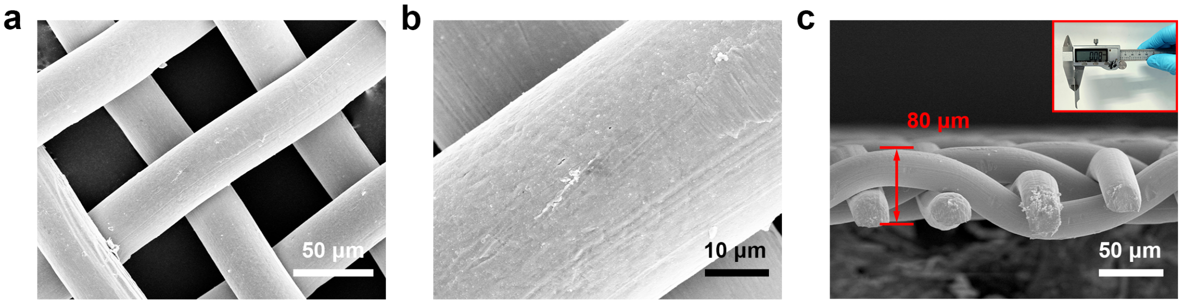

Supplementary Figure 4. a, Top image and **b,** high-magnification image of the SSM substrate. The wire spacing is ~ 50 µm, and the diameter of the individual wire is ~ 30 µm. c, The cross-sectional SEM image and the corresponding vernier caliper measurement of the SSM substrate, indicating that the membrane thickness is ~ 80 µm.

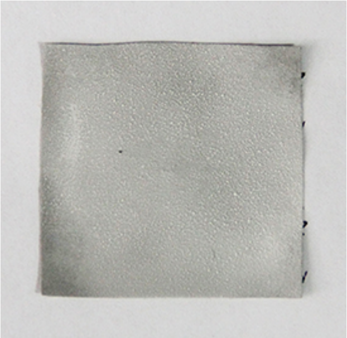

Supplementary Figure 5. Digital picture of FS-SSM with a rough surface.

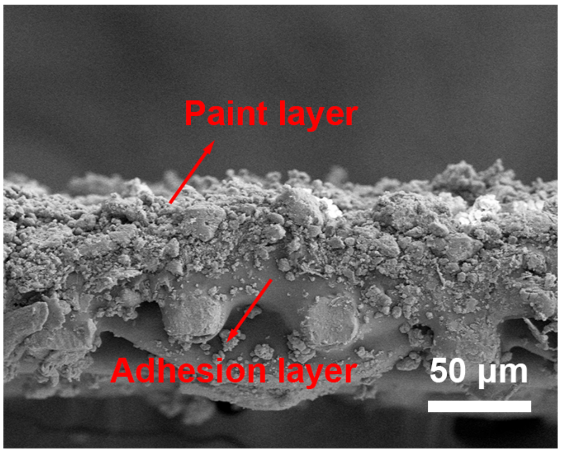

Supplementary Figure 6. The cross-sectional SEM images of the FS-SSM membrane. The two superimposed layers can be clearly observed that contains the paint layer and the adhesion layer.

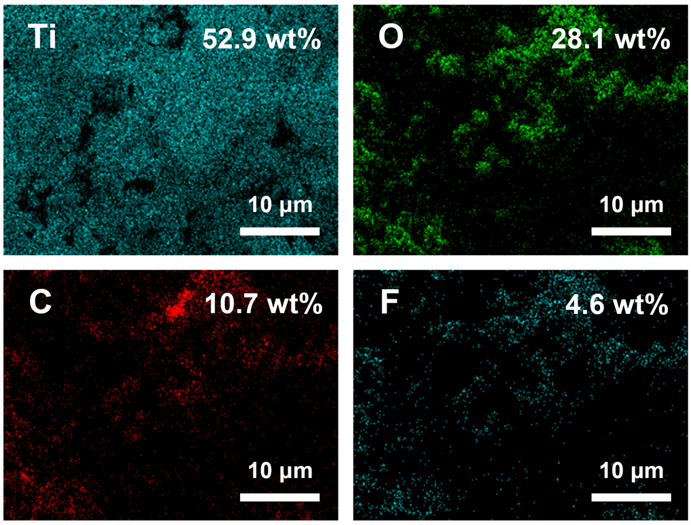

Supplementary Figure 7. Atomic concentration of FS-SSM. The EDX mapping clearly discerns the additional presence of Ti and F elements compared with the SSM substrate.

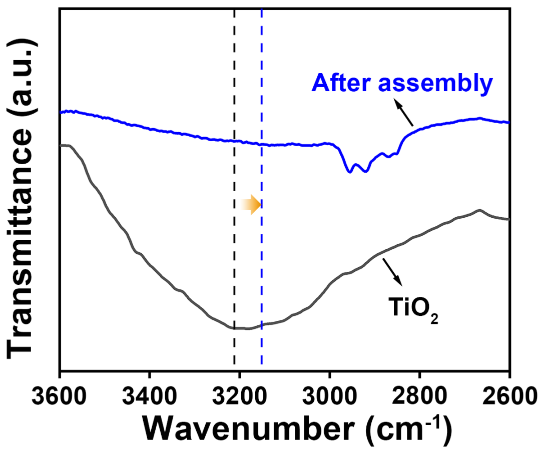

Supplementary Figure 8. The red shift of the hydroxyl group on the surface of TiO_2. Typically, the stretching vibration peak of the hydroxyl group on the surface of TiO_2 nanoparticles is at 3212 cm^{-1}. Whereas the red shift phenomenon is performed for the as-prepared membrane that the corresponding peak of the hydroxyl group adjusts to 3151 cm^{-1}. The change verifies the occurrence of the hydrogen bond self-assembly between fluorosurfactant and TiO_2 nanoparticles, which transforms the electronic cloud density of the O-H bond.

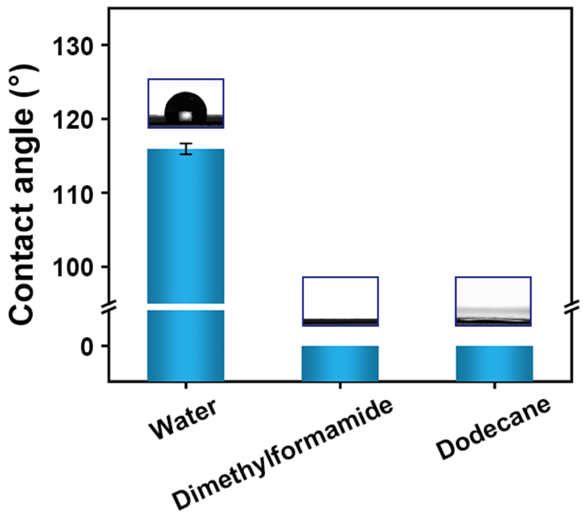

Supplementary Figure 9. The contact angles of water, dimethylformamide, and dodecane on the SSM substrate. The substrate exhibits different wettability for the typical OLs compared with FS-SSM, suggesting the coated composite layers play a crucial role in the final unique wetting behaviors for various liquids rather than the SSM substrate.

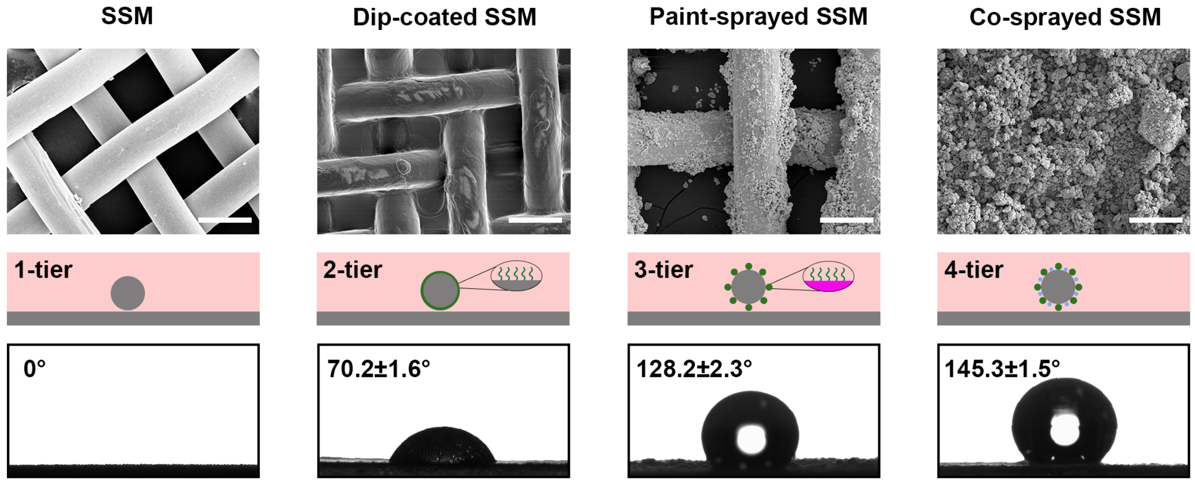

Supplementary Figure 10. Hierarchical tiers of the fabricated surfaces and the corresponding wettability. Based on the SEM images (scale bar is 50 μm) and corresponding schematics, the mesh substrate possesses one tier of surface hierarchy compared with the smooth substrate. The amphoteric fluorosurfactant is dip-coated on the mesh to realize two tiers of surface hierarchies. Three tiers of surface hierarchies are further prepared by the spray-coating method with the paint only. Whereas with the "glue + paint" co-spray-coating method, the FS-SSM features the four tiers of surface hierarchies which significantly reduces the liquid-solid contact area. As exhibited in the contact angle profiles, the increased tiers of hierarchies can promote the repellence to the probe liquid (cyclohexane), indicating the crucial role of the multi-re-entrant hierarchical structure.

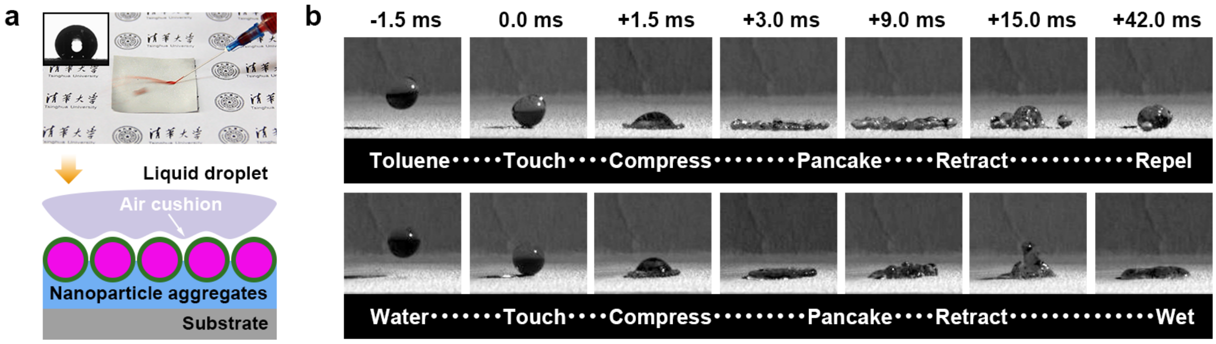

Supplementary Figure 11. The rapid response of different wetting behaviors of the FS-SSM. **a,** Images illustrate a jet of toluene bouncing off the membrane surface without residual liquid based on the proposed explanation of low-adhesion quasi-superlyophobic surface. **b,** Time-sequence photographs of toluene droplet bouncing and water wetting on the membrane (the contact moment of the liquid droplet and the solid surface is defined as 0). The nonpolar toluene droplet bounced up in a short time without wetting the surface, indicating the weak interaction between toluene and FS-SSM. On the contrary, the water droplet spread rapidly on the surface due to the strong affinity. Droplet sizes, ~ 6 μL.

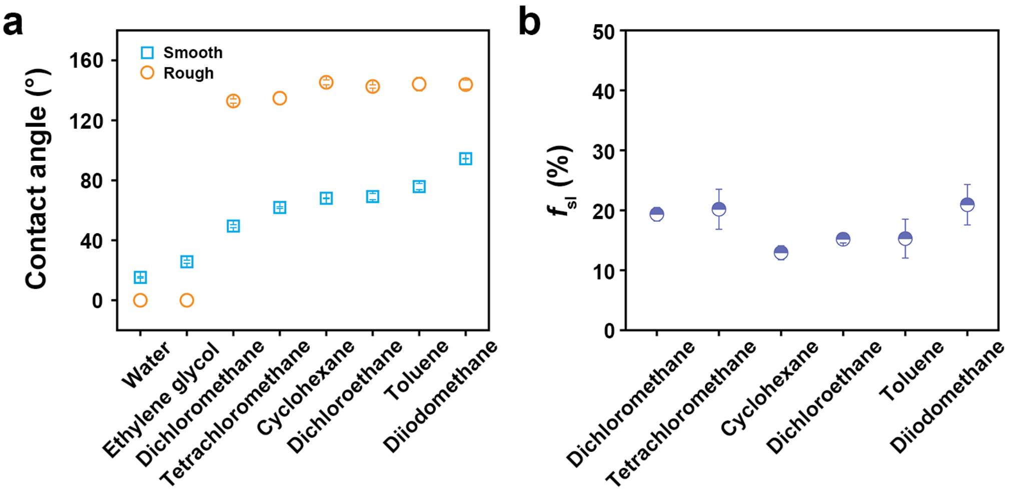

Supplementary Figure 12. The wetting performance of the FS-SSM. **a,** The contact angles of various OLs on the as-prepared membrane and the corresponding smooth surface. **b,** The calculational values of f_{sl} based on the intrinsic contact angle and the enhanced contact angle of different liquids in Cassie-Baxter state. It can be seen that the values are distributed in a small range, indicating the accuracy of the calculational results.

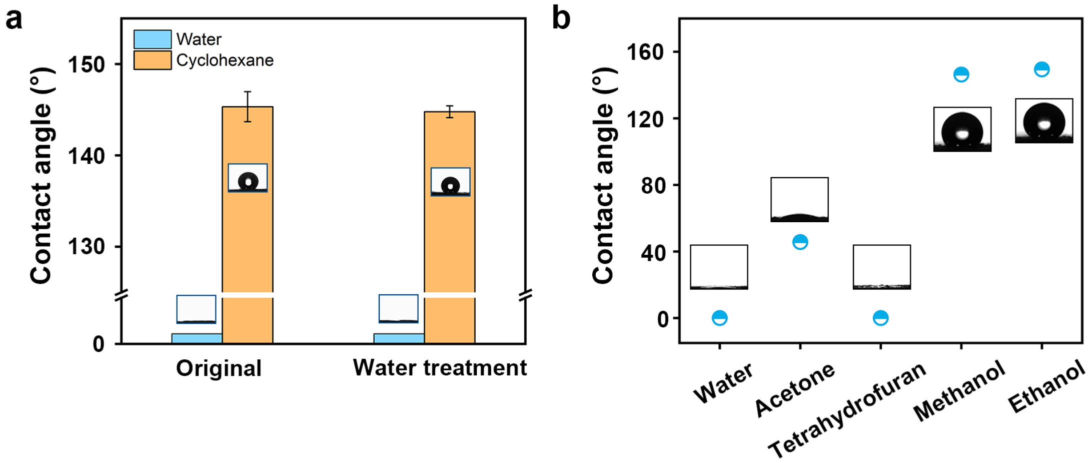

Supplementary Figure 13. The water-tolerant stability and wettability transition of the FS-SSM. **a,** The contact angles of the probe liquids (cyclohexane and water) on the FS-SSM before and after the water immersion treatment (1 h). It can be seen that the membrane wettability is nearly unchanged under the corresponding treatment compared with the original. Although the Capstone FS-50 is one type of the amphoteric fluorosurfactant that can be slightly soluble in water, the membrane possesses excellent chemical stability due to the strong hydrogen bond and mechanical interlocking interactions of the proposed layers. **b,** The wettability of the surface coating after different wetting liquids immersion. To explore the wettability switching property of the coating, the as-prepared membranes were immersed in different solvents for 5 min, respectively. Water is selected as the probe liquid to test the corresponding contact angles. After the treatment of filtering liquids (the passing phase in the subsequent separation process, water as the tested example) or other wetting liquids (medium polar solvents that can be miscible with most OLs, tetrahydrofuran as the tested example), the surface still exhibits the same superhydrophilicity as before (details in Supplementary Table 1). In contrast, the superhydrophobicity that is the reverse mode compared with the initial state can be conducted after the treatment of methanol and ethanol (strong polar solvents that are miscible with most OLs). It can be seen that ethanol can induce a better changing extent, which is used as the representative joystick liquid in this work.

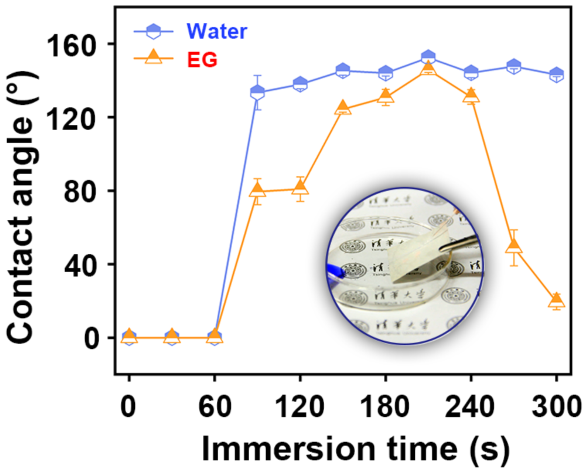

Supplementary Figure 14. Contact angles of water and ethylene glycol (EG) on the surface as a function of immersion time in ethanol. The inset shows the repellency of the chosen PR-SSM to water flowing.

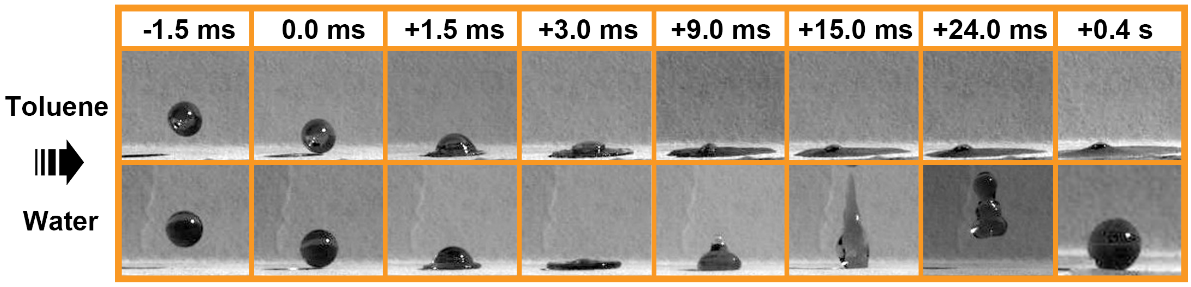

Supplementary Figure 15. Time-sequence photographs of toluene droplet bouncing and water wetting on the PR-SSM. Droplet sizes, ~ 6 μL. The water droplet bounces some times before residing on the surface, indicating the weak interaction between water and PR-SSM. On the contrary, toluene droplet spreads rapidly on the surface due to its strong affinity.

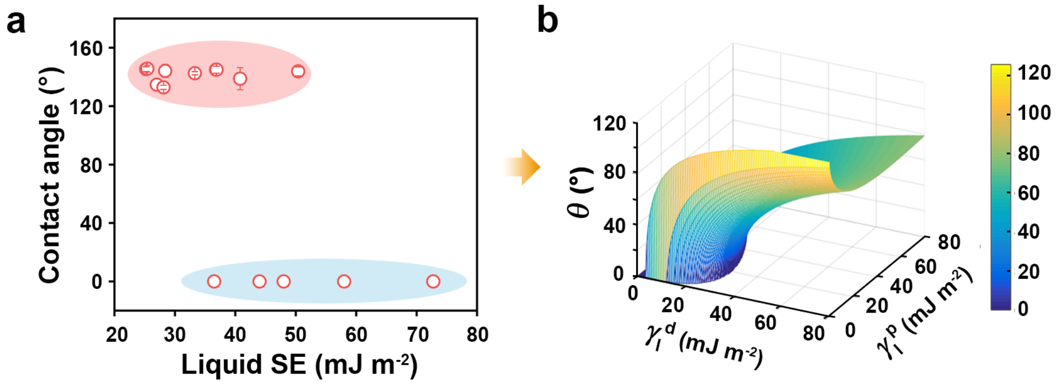

Supplementary Figure 16. Dependence of the wettability on the liquid SE or the corresponding components. **a,** The relationship between the SE and the corresponding contact angle of various liquids on the membrane. **b,** θ as a function of γ_l^d and γ_l^p for manipulating OLs action.

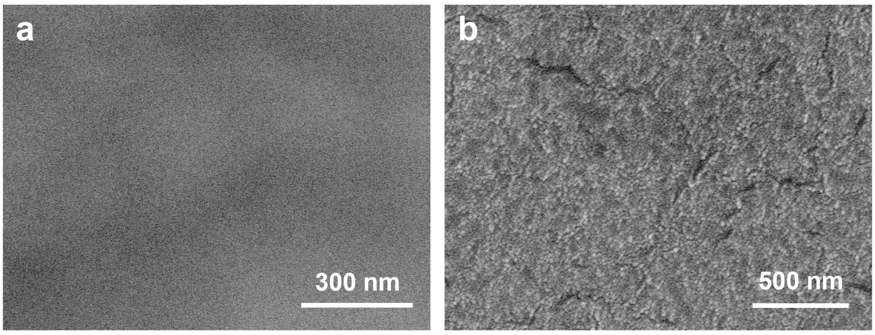

Supplementary Figure 17. SEM images of the flat samples before and after the trigger. **a,** SEM image of the flat silicon wafer coated Capstone FS-50. This surface is an ideal smooth one that can be used as the intrinsic state of FS-SSM. **b,** SEM image of the flat silicon wafer as the intrinsic state of PR-SSM. The sample can be regarded as a model to explore the intrinsic wetting behaviors of liquids on the PR-SSM due to the significantly smoother morphology compared with the membrane.

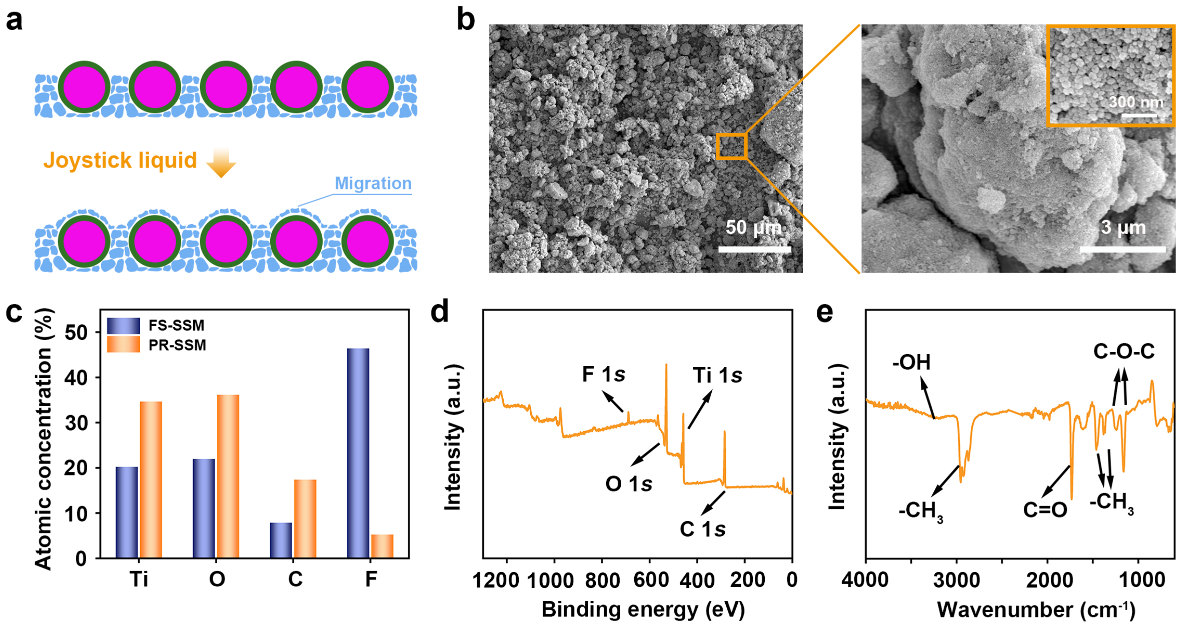

Supplementary Figure 18. The characterization of surface morphology and chemical composition of the PR-SSM. **a,** Schematic illustration of the achievement of the opposite wetting behaviors through particle rearrangement. **b,** SEM images of the PR-SSM. The microstructure identifies the reassembly result of TiO_2 nanoparticles intuitively, as evidenced by the produced larger scale of spherical nanoparticle aggregation after the treatment of ethanol. **c,** Variation of element content between FS-SSM and PR-SSM. Compared with the original FS-SSM, increased carbon element and a sharp decrease of the fluorine element are detected to be distributed on the surface of PR-SSM, highlighting the exposure of glue molecules on the nanoparticles' interfaces. **d,** XPS wide spectra of PR-SSM. The increased C signal and decreased F signal of the membrane after the ethanol trigger convincingly proves the process of particle rearrangement. **e,** FTIR spectra of PR-SSM. The characteristic peaks of the PR-SSM coincide with the corresponding peaks of G-SSM, meaning that polyacrylate structure can be strongly detected on account of the migration process of glue molecules.

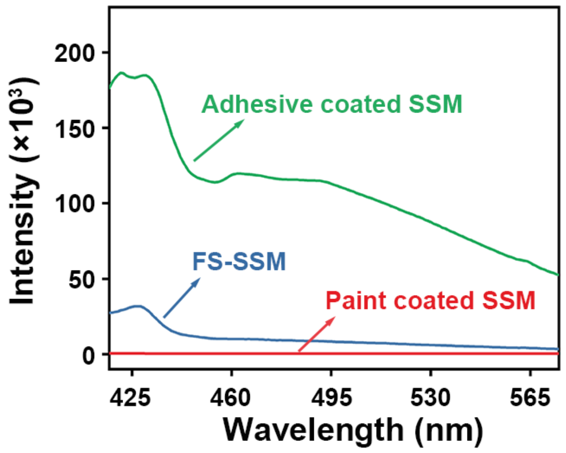

Supplementary Figure 19. Fluorescence spectra of different membranes including the adhesive coated SSM, paint coated SSM, and the final FS-SSM (λ_{ex} = 405 nm). The used polyacrylate adhesive exhibits strong blue fluorescence (420 ~ 450 nm), while no fluorescence is recorded with the paint nanoparticles.

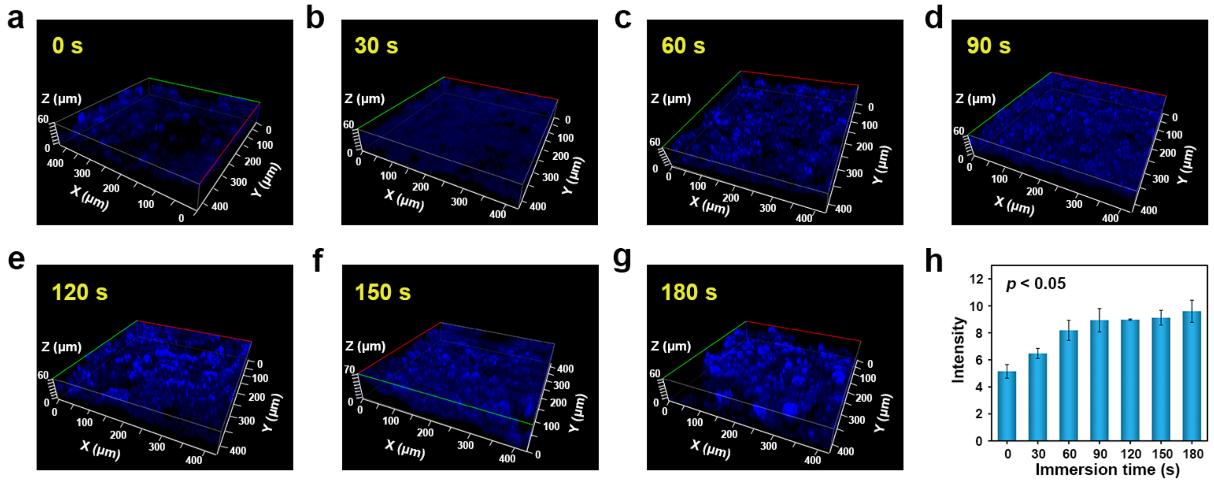

Supplementary Figure 20. Demonstration of the dynamic transition process of the FS-SSM surface via joystick liquid triggering. **a-g,** The confocal images of the FS-SSM after different treatment time of the joystick liquid (30 s interval). "0 s" and "180 s" represents the stages of FS-SSM and PR-SSM, respectively. Each sample was tested on three different positions and taken the average (λ_{ex} = 405 nm). **h,** Fluorescent intensities of the FS-SSM after different treatment time of the joystick liquid, $p < 0.05$ between the initial membrane and other states, data are represented as mean ± standard deviation (SD).

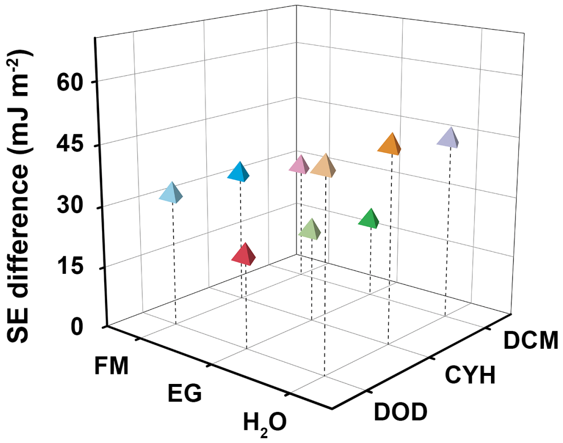

Supplementary Figure 21. The total SE differences of the nine OL pairs. These liquid mixtures can be efficiently and controllably separated by the reconfigurable membrane.

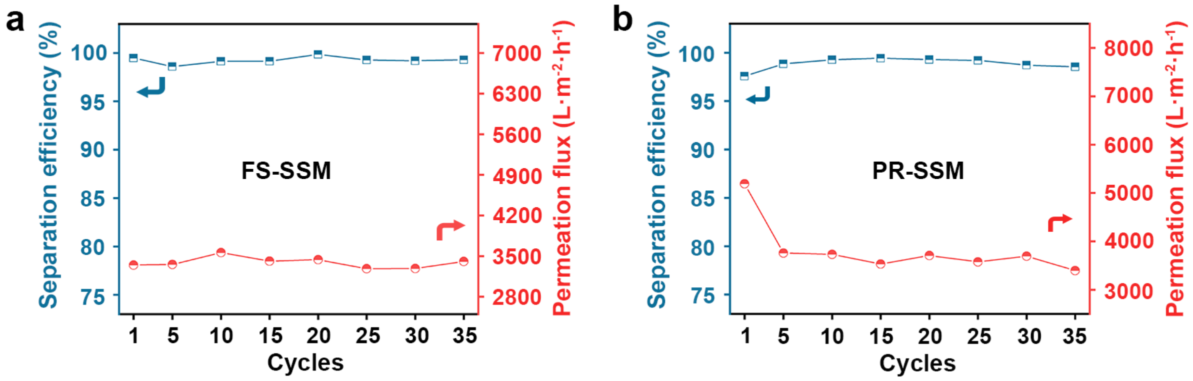

Supplementary Figure 22. The cycle stability experiments of the FS-SSM and PR-SSM. **a,** To assess the long-term reusability of the FS-SSM, cycle stability experiments were performed for 35 cycles, taking formamide/dodecane mixture as the representative sample. On the one hand, the as-prepared mesh exhibits a stable permeation flux with no significant decline during 35 cycles, demonstrating that the as-prepared membrane is equipped with an antifouling property. On the other hand, high separation efficiency and low insoluble phase residual remained in separation cycles, exhibiting excellent durability in long-term work. **b,** Change of the separation efficiency and permeation flux of the PR-SSM as separating formamide/dodecane mixture for 35 cycles. The membrane shows superior separation efficiency (> 98.5%) during the continuous separation process. Moreover, the permeation flux is still above 3,400 L m^{-2} h^{-1} after 35 cycles, indicating superior cycle stability of the membranes.

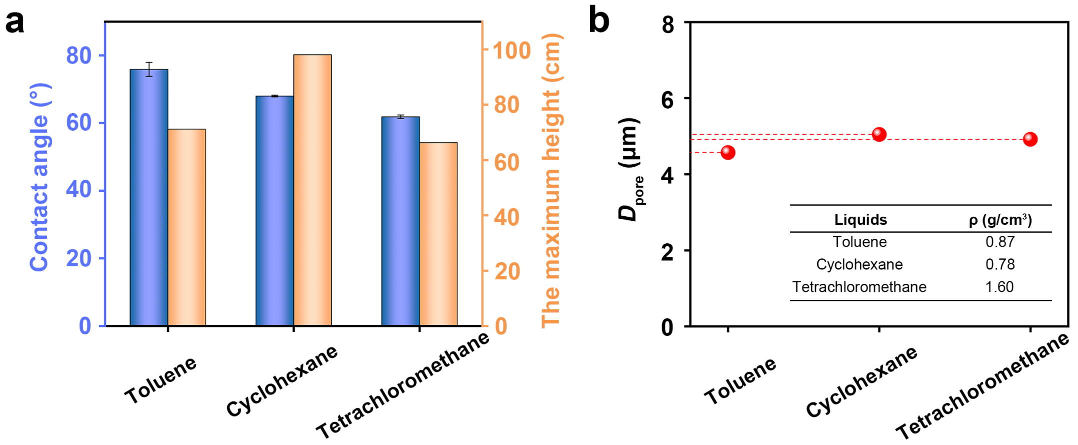

Supplementary Figure 23. Calculational average pore diameter of the membrane based on intrusion pressure tests. **a,** The contact angles of liquids on the flat solid surface and the maximum height of liquids that the FS-SSM can support. The intrinsic surface exhibits lyophobicity for all three liquids (above the wetting threshold, about 65°), and the FS-SSM possesses the excellent durability for intruding pressure of different liquids. **b,** The calculational pore diameter of the FS-SSM. D_{pore} can be obtained according to the liquid pressure test and the corresponding liquid densities of toluene, cyclohexane, and tetrachloromethane. It can be seen that the values are distributed in a small range, indicating the accuracy of the calculational results.

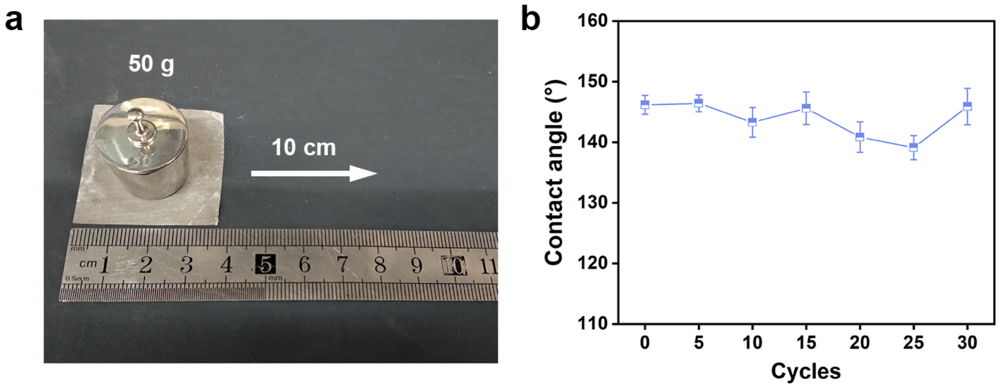

Supplementary Figure 24. Sandpaper abrasion tests of the FS-SSM. **a,** One cycle of the sandpaper abrasion test. The membrane is pressed by a 50 g weight and placed face-down to the sandpaper (standard abrasive paper, CW 1,200). The membrane sample is dragged for 10 cm along the ruler with a speed of 3 cm/s. This process is defined as one abrasion cycle. **b,** The variation of cyclohexane contact angles during 30 mechanical abrasion cycles. It can be observed that the contact angles remain static, indicating the FS-SSM possesses outstanding mechanical durability.

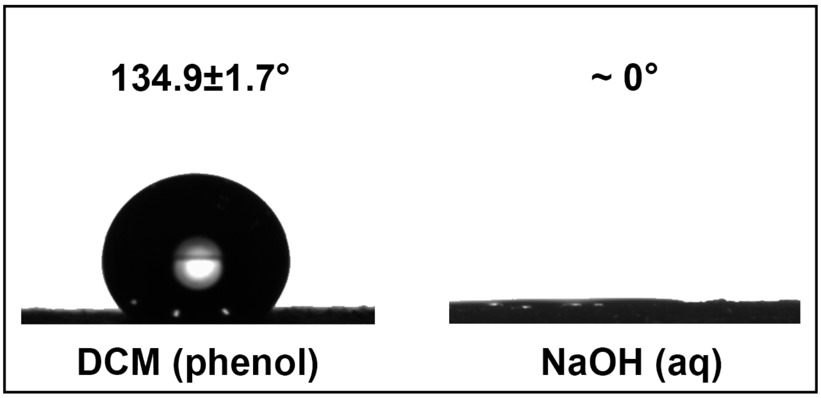

Supplementary Figure 25. The contact angles of the DCM solution of phenol (500 ppm) and 10 wt% NaOH aqueous solution on the FS-SSM. The FS-SSM exhibits the high-lyophobicity for DCM solution of phenol but superlyophilicity for NaOH aqueous solution, manifesting the feasibility of in-situ back extraction.

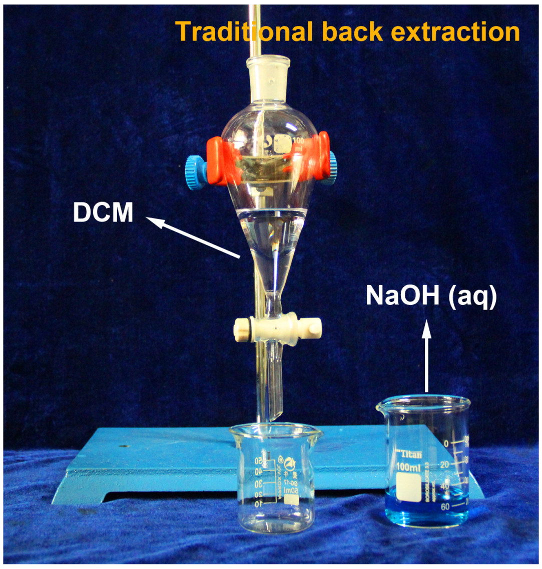

Supplementary Figure 26. Photograph of the traditional back extraction operation. The traditional method needs more vessels, longer operating time, and more complicated procedures compared with in situ back extraction of the FS-SSM.

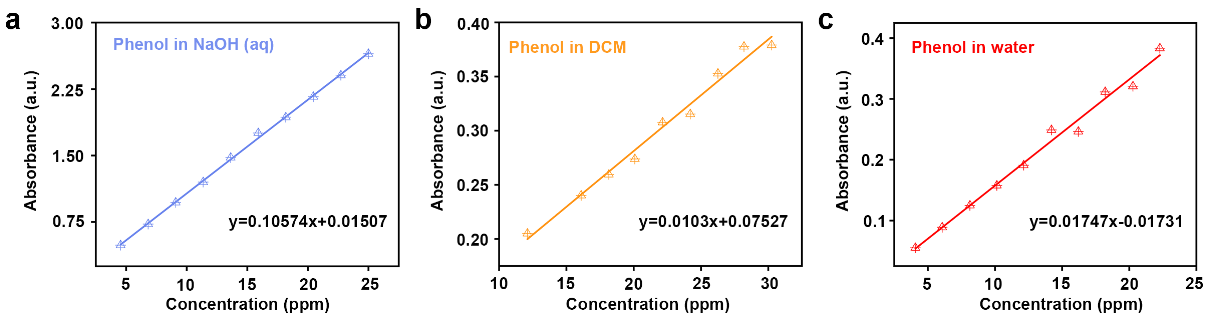

Supplementary Figure 27. Standard curves of concentration of phenol in different solutions. **a,** Standard curve of concentration of phenol in 10 wt% NaOH (aq) for UV-vis spectra. A series of standard samples (10 wt% NaOH aqueous solution containing phenol) with known concentrations are analyzed, and then the standard curves are calculated by correlating the concentration with absorbance. **b,** Standard curve of concentration of phenol in DCM for UV-vis spectra. A series of standard samples (DCM solution containing phenol) with known concentrations are analyzed, and then the standard curves are calculated by correlating the concentration with absorbance. **c,** Standard curve of concentration of phenol in water for UV-vis spectra. A series of standard samples (aqueous phenol solution) with known concentrations are analyzed, and then the standard curves are calculated by correlating the concentration with absorbance.

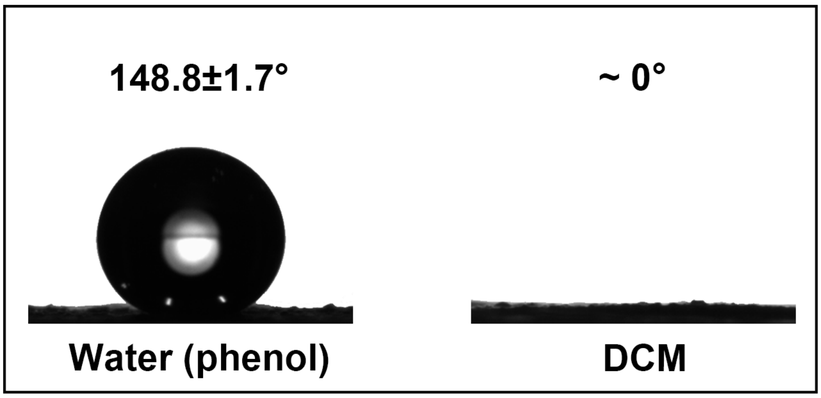

Supplementary Figure 28. The contact angles of the aqueous solution of phenol (500 ppm) and DCM on the PR-SSM. The PR-SSM exhibits the high-lyophobicity for aqueous phenol solution but superlyophilicity for DCM, manifesting the feasibility of in-situ extraction.

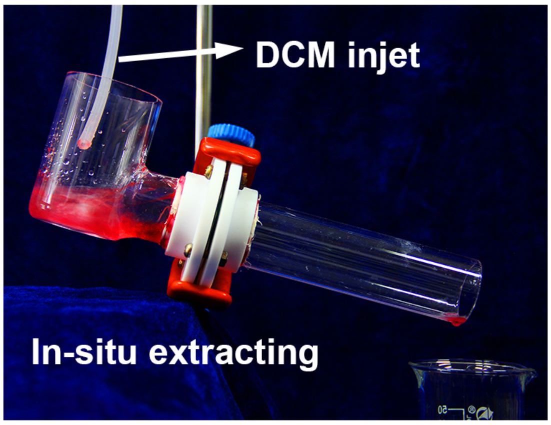

Supplementary Figure 29. Photograph of in situ extraction process based on PR-SSM. PR-SSM is assembled in the extraction device, and constant feed of DCM (extraction agent dyed in red) is carried out under strong stirring. During the whole process, DCM that contains the phenol flows out the membrane to achieve the real-time in situ extraction.

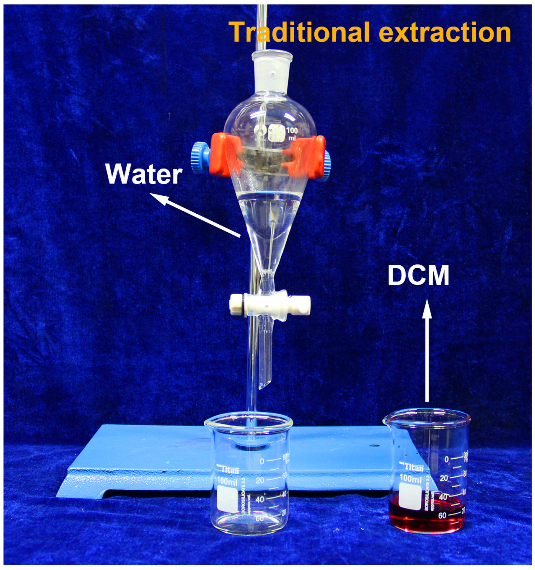

Supplementary Figure 30. Photograph of the traditional extraction operation. This route requires multiple procedures of mixing, transferring, centrifuging, and waiting for phase equilibrium.

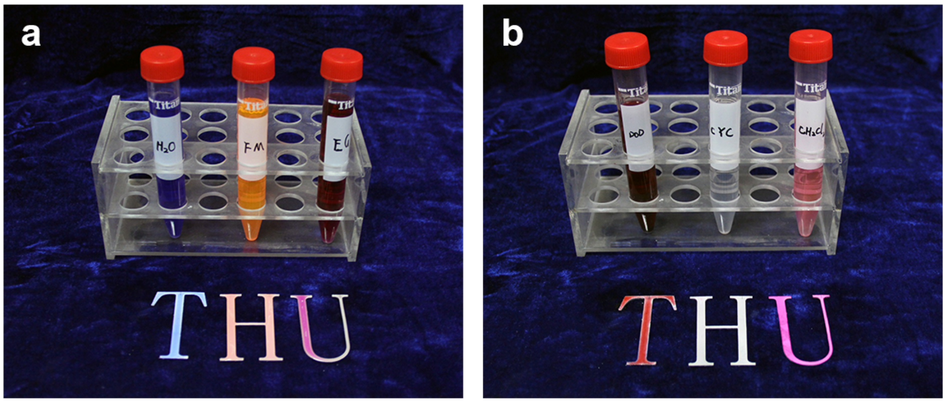

Supplementary Figure 31. Pattern “THU” deriving from the smart coating. **a,** To take advantage of the described wetting phenomenon, we applied a designed mold to create superlyophilic patterns with different colors on the flat stainless steel surface. For the original state of the co-spray-coated pattern, the coated parts exhibit the superlyophilicity to water, formamide (FM), and ethylene glycol (EG). **b,** After the treatment of the ethanol, the corresponding parts show the superlyophilicity to dodecane (DOD), cyclohexane (CYH), and dichloromethane (CH_2Cl_2).

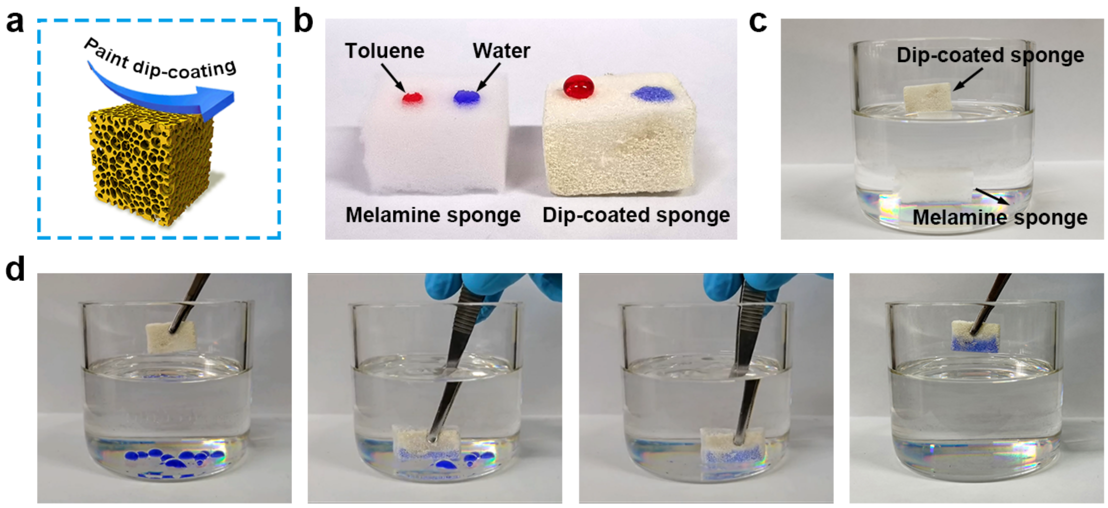

Supplementary Figure 32. The purification of organic reagents via a paint modified sponge. **a,** Schematic illustration of the fabrication of the superwetting sponge as liquid absorbent. **b,** Because of the amphiphilicity, the pristine melamine sponge absorbs both toluene (dyed in red) and water (dyed in blue) drops, making them unsuitable for selective removal of heterogeneous water phase from the toluene/water mixture. The paint dip-coating treatment endows the melamine sponge with the high-lyophobicity to toluene and the superlyophilicity to water. **c,** Due to the aforementioned wettability, the dip-coated sponge readily floated on the toluene surface as opposed to the immediate sinking of the pristine sample. **d,** The dip-coated sponge could be used as an ideal absorbent for purifying or recycling the organic reagent based on its special wettability and high porosity. Here a small piece of dip-coated sponge is placed in the bottom of the toluene/water mixture, and it could selectively absorb the water phase (dyed in blue), leaving behind the clean toluene phase.

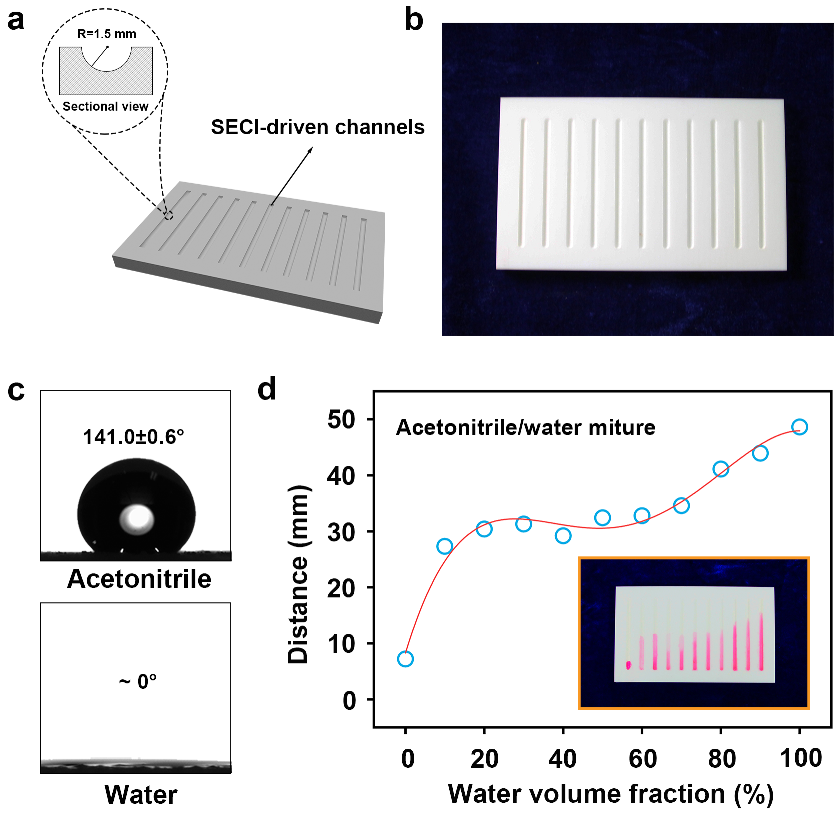

Supplementary Figure 33. Composition Measurement of the binary miscible mixture (acetonitrile/water mixture as the example). **a,** A schematic highlighting the design of a water meter. **b,** The device is made of PTFE with a set of parallel grooves coated with the adhesive and the as-prepared paint. Varying composition of the miscible OL-water mixtures which possess different SECI can generate discrepant wetting length on the channels. We can determine the composition of the unknown miscible mixture according to the corresponding wetting length. **c,** The wetting behaviors of acetonitrile and water on the coated PTFE substrate. **d,** The standard-fit curve of wetting lengths of acetonitrile-water mixtures with known compositions. The inset is the measurement of the composition of miscible OL mixtures with a SECI-driven device. The photograph exhibits the different wetting cases of acetonitrile-water mixtures (the mixtures dyed in pink).

Supplementary Tables

Supplementary Table 1. Summary of the effect of wetting OLs on the FS-SSM surface.

Liquid[a)]	Membrane behavior[b)]	Membrane wettability	Trigger[c)]
Water	Unchanged	Stable	No
Formamide	Unchanged	Stable	No
Ethylene glycol	Unchanged	Stable	No
Dimethyl sulfoxide	Unchanged	Stable	No
Dimethylformamide	Unchanged	Stable	No
Methanol	Swelling	Inverse	Yes
Ethanol	Swelling	Inverse	Yes
Acetone	Slightly swelling	Metastable	No
Tetrahydrofuran	Unchanged	Stable	No

[a)] The FS-SSM is immersed for 5 min in these liquids that exhibit lyophilic properties to the membrane; [b)] The surface morphology states of the membrane after the corresponding treatments; [c)] The determination of the joystick liquids.

Supplementary Table 2. SE components of the targeted surfaces.

Membrane	γ_s[a)] (mJ m^{-2})	γ_s^d (mJ m^{-2})	γ_s^p (mJ m^{-2})
FS-SSM	80.91	3.56	77.35
PR-SSM	26.79	24.57	2.22

[a)] The total SE (γ_s) and the corresponding components (γ_s^d and γ_s^p) of the membranes are measured by Fowkes method (Supplementary Note 3).

Supplementary Table 3. Components of SE and SECI for various liquids.

Liquid[a)]	γ_l[b)] (mJ m^{-2})	γ_l^d (mJ m^{-2})	γ_l^p (mJ m^{-2})	Ψ^d (mJ$^{-1/2}$ m)	Ψ^p (mJ$^{-1/2}$ m)
Water	72.8	22.1	50.7	0.0646	0.0978
Formamide	58.0	39.0	19.0	0.1077	0.0752
Diiodomethane	50.4	49.1	1.3	0.1390	0.0226
Ethylene glycol	48.0	29.0	19.0	0.1122	0.0908
Dimethyl sulfoxide	44.0	36.0	8.0	0.1364	0.0643
Nitromethane	36.8	29.8	7.0	0.1483	0.0719
Dimethylformamide	36.5	25.2	11.3	0.1375	0.0921
Dichloroethane	33.3	30.8	2.5	0.1667	0.0475
Toluene	28.4	26.1	2.3	0.1799	0.0534
Tetrachloromethane	27.0	26.7	0.3	0.1914	0.0203
Cyclohexane	25.2	25.2	0.0	0.2331	0.0000

[a)] The liquids are sorted by the values of total SE (γ_l); [b)] SE values with the components (at 25°C if unspecified) were obtained from the literature. The values of Ψ^p and Ψ^d are calculated in detail based on Supplementary Equation 15 and 16 in Supplementary Note 2, and the corresponding unit is adjusted to mJ$^{-1/2}$ m.

Supplementary Table 4. Relationship between liquid wettability and SE or polar SECI of the FS-SSM.

Liquid[a)]	γ_l (mJ m^{-2})	Ψ^p (mJ$^{-1/2}$ m)	Contact angle (°)
Cyclohexane	25.2	0.0000	145.3±1.6
Tetrachloromethane	27.0	0.0203	134.8±3.9
Diiodomethane	50.4	0.0226	143.9±3.0
Dichloroethane	33.3	0.0475	142.6±1.1
Toluene	28.4	0.0534	144.2±4.5
Dimethyl sulfoxide	44.0	0.0643	0
Nitromethane	36.8	0.0719	145.0±2.4
Formamide	58.0	0.0752	0
Ethylene glycol	48.0	0.0908	0
Dimethylformamide	36.5	0.0921	0
Water	72.8	0.0978	0

[a)] The liquids are sorted by the values of Ψ^p, and each data matches Fig. 3c.

Supplementary Table 5. Relationship between liquid wettability and SE or dispersive SECI of the PR-SSM.

Liquid[a)]	γ_l (mJ m^{-2})	Ψ^d (mJ$^{-1/2}$ m)	Contact angle (°)
Water	72.8	0.0646	152.4±3.1
Formamide	58.0	0.1077	147.8±2.3
Ethylene glycol	48.0	0.1122	145.8±1.5
Dimethyl sulfoxide	44.0	0.1364	141.5±5.5
Dimethylformamide	36.5	0.1375	142.2±3.1
Diiodomethane	50.4	0.1390	144.2±4.9
Nitromethane	36.8	0.1483	139.7±1.1
Dichloroethane	33.3	0.1667	60.3±6.9
Toluene	28.4	0.1799	0
Tetrachloromethane	27.0	0.1914	0
Cyclohexane	25.2	0.2331	0

[a)] The liquids are sorted by the values of Ψ^d, and each data matches Fig. 3e.

Supplementary Table 6. Summary of the OL pairs that can be separated controllably with the smart system.

Pairs[a)]	H_2O	FM	EG	DOD	CYH	DCM	DMF	DMSO	TL	DIM	CCl_4
CCl_4	Dual[b)]	Dual	Dual	—	—	—	—	—	—	—	—
DIM	Single[c)]	Single	Single	—	—	—	—	—	—	—	—
TL	Dual	Dual	Dual	—	—	—	—	—	—	—	—
DMSO	—[d)]	—	—	Dual	Dual	—	—	—	—	—	—
DMF	—	—	—	Dual	Dual	—	—	—	—	—	—
DCM	Dual	Dual	Dual	—	—	—	—	—	—	—	—
CYH	Dual	Dual	Dual	—	—	—	Dual	Dual	—	—	—
DOD	Dual	Dual	Dual	—	—	—	Dual	Dual	—	—	—
EG	—	—	—	Dual	Dual	Dual	—	—	Dual	Single	Dual
FM	—	—	—	Dual	Dual	Dual	—	—	Dual	Single	Dual
H_2O	—	—	—	Dual	Dual	Dual	—	—	Dual	Single	Dual

[a)] The liquid mixtures that have been separated via the membranes. FM: formamide; EG: ethylene glycol; DOD: dodecane; CYH: cyclohexane; DCM: dichloromethane; DMF: dimethylformamide; DMSO: Dimethyl sulfoxide; TL: Toluene; DIM: Diiodomethane; [b)] "Dual" indicates the corresponding immiscible mixtures can be controllably separated; [c)] "Single" indicates the FS-SSM can only separate the liquid pairs and PR-SSM is invalid due to the amphiphobicity in this case; [d)] Symbol "—" indicates that the corresponding two liquids are miscible and inseparable.

Supplementary Table 7. Summary of the abbreviations used in this work.

Abbreviations	Full name
OL	Organic liquid
SE	Surface energy
SECI	Surface energy component index
SSM	Stainless steel mesh
G-SSM	Glue coated SSM
FS-SSM	The as-prepared membrane
PR-SSM	The membrane after the joystick liquid response
DOD	Dodecane
CYH	Cyclohexane
DCM	Dichloromethane
FM	Formamide
EG	Ethylene glycol

Supplementary Notes

Supplementary Note 1. The recursive theory underlying the role of the hierarchical structure.

In order to explore the crucial role of the elaborated microstructure, a recursion method is performed based on the Cassie-Baxter equation.[2-6] The derived formulas in different cases are exhibited below.

For the surface featured with arranged nanoparticles:

$$\cos\theta^*_{\text{particle}} = -1 + \frac{1}{d^{*2}}\left[\frac{\pi}{4}(1+\cos\theta)^2\right] \quad (1)$$

While for the surface composed of periodic cylinders:

$$\cos\theta^*_{\text{wire}} = -1 + \frac{1}{d^*}\left[\sin\theta + (\pi\text{-}\theta)\cos\theta\right] \quad (2)$$

Where d^* represents the linear spacing ratio of the surface texture ($d^* = 1 + D/R$, R represents the mean radius of the nanoparticle or periodic cylinders, D is the mean radius of interspacing).

First, the liquid wetting behavior on the smooth surface ($d_1^* = 1$) can be described by the aforementioned Young's equation:

$$\cos\theta_1 = \frac{\gamma_{\text{s}} - \gamma_{\text{sl}}}{\gamma_{\text{l}}} \quad (3)$$

For the particle surface with 2-tier hierarchical structure ($d_2^* = d_{\text{particle}}^* \times d_1^*$), Cassie-Baxter equation can be rewritten as:

$$\cos\theta_2 = -1 + \frac{1}{d_2^{*2}}\left[\frac{\pi}{4}(1+\cos\theta_1)^2\right] \quad (4)$$

By introducing the polyacrylate adhesive in our case, the above particles would aggregate to form the sophisticated surface with 3-tier hierarchical structure ($d_3^* = d_{\text{aggregate}}^* \times d_2^* \times d_1^*$), leading to the following equation:

$$\cos\theta_3 = -1 + \frac{1}{d_3^{*2}}\left[\frac{\pi}{4}(1+\cos\theta_2)^2\right] \quad (5)$$

If such aggregates are formed on the mesh substrate ($d_4^* = d_{wire}^* \times d_3^* \times d_2^* \times d_1^*$), we can obtain 4-tier hierarchical structure, and the final formulation becomes as:

$$\cos\theta_4 = -1 + \frac{1}{d_4^*}\left[\sin\theta_3 + (\pi\text{-}\theta_3)\cos\theta_3\right] \tag{6}$$

Based on the above derivation, it can be seen that the hierarchical structure is crucial to the extent of the liquid repellence. Supplementary Fig. 10 further verifies the above results. Owing to the fabricated 4-tier hierarchical structure and unique SE components, the as-prepared surface exhibits quasi-superlyophobicity and low adhesive performance for these liquids (Supplementary Fig. 11a). Such a surface brought a rapid response of different wetting behaviors verified by the corresponding bounce tests (Supplementary Fig. 11b). When a liquid droplet sits on the surface composed of such nanoparticle aggregates in Cassie-Baxter state, the apparent contact angle (θ^*) on the surface can be evaluated as:[2,7]

$$\cos\theta^* = f_{sl}\cos\theta - 1 + f_{sl} \tag{7}$$

Where f_{sl} is the fraction of the projected area of the solid surface in contact with the liquid. Considering the intrinsic contact angle and the enhanced contact angle of different liquids, the f_{sl} is statistically calculated to be as low as 17.3% (Supplementary Fig. 12). Thus, the nanoscale surface roughness can minimize the interacting area between droplet and surface structure, guaranteeing a continuous air layer for repelling the liquid.

Supplementary Note 2. Derivation process of SECI parameter.

Thus far, most of the existing superwetting membranes involving OL separation exhibit generally recognized behaviors that lower SE liquids flow through whereas hampering higher SE liquids, which are consistent with the thumb-up rule of Young's equation:[8,9]

$$\cos\theta = \frac{\gamma_s - \gamma_{sl}}{\gamma_l} \tag{8}$$

Where θ is the equilibrium contact angle of the liquid on the flat solid surface at the three-phase contact line; γ_s, γ_l, and γ_{sl} represent the surface energies of solid, liquid, and solid-liquid, respectively. Despite the prevalent wetting phenomenon that can be well explained by comparing the total SEs of solids and liquids,[10-12] it is not efficient enough to characterize the contact status of various OLs. From the surface chemistry perspective, the interaction between solid and liquid results from various types of well-known molecular forces, such as London dispersion forces and hydrogen bonding.[13-19] As postulated by Fowkes in the interfacial thermodynamics theory, the SE components can be divided into a dispersive part and a polar part that can be treated independently with neglecting the polar-dispersive interactions:[13,14]

$$\gamma_{sl} = \gamma_s + \gamma_l - 2\sqrt{\gamma_s^d \gamma_l^d} - 2\sqrt{\gamma_s^p \gamma_l^p} \tag{9}$$

Where the superscripts d and p refer to the dispersion force and polar force components, respectively. Owens, Wendt, Rabel, and Kaelble theory (OWEK) relates different surface energy of two phases with the corresponding contact angles as followed below:[15]

$$\gamma_l(1+\cos\theta)=2\sqrt{\gamma_s^d \gamma_l^d} + 2\sqrt{\gamma_s^p \gamma_l^p} \tag{10}$$

According to Supplementary Equation 10, both of the SE components are suggested to be efficient parameters to influence the wetting behaviors of liquids on the solid surface. However, until now, there are few works focused on the quantitative relation between the physical properties for guiding the on-demand fabrication of superwetting membranes logically. Therefore, it is critical to building up a feasible methodology to tailor the appropriate surface

chemistry using basic liquid properties effectively. Based on the separation of variables, Supplementary Equation 10 can be changed in the following form:

$$\begin{aligned}\cos\theta &= \frac{2\sqrt{\gamma_s^d \gamma_l^d} + 2\sqrt{\gamma_s^p \gamma_l^p}}{\gamma_l^d + \gamma_l^p} - 1 \\ &= 2\sqrt{\gamma_s^d}\sqrt{\frac{\gamma_l^d}{(\gamma_l^d + \gamma_l^p)^2}} + 2\sqrt{\gamma_s^p}\sqrt{\frac{\gamma_l^p}{(\gamma_l^d + \gamma_l^p)^2}} - 1 \\ &= 2\sqrt{\gamma_s^d}\sqrt{\frac{\varphi_l^d}{\gamma_l^d + \gamma_l^p}} + 2\sqrt{\gamma_s^p}\sqrt{\frac{\varphi_l^p}{\gamma_l^d + \gamma_l^p}} - 1\end{aligned} \tag{11}$$

Thereinto, to intuitively express the relationship between liquid parameters, we set φ_l^d and φ_l^p as the proportion of dispersive and polar parts in total liquid SE:

$$\varphi_l^d = \frac{\gamma_l^d}{\gamma_l^d + \gamma_l^p} \tag{12}$$

$$\varphi_l^p = \frac{\gamma_l^p}{\gamma_l^d + \gamma_l^p} \tag{13}$$

The deuterogenic Supplementary Equation 11 implies that arbitrary liquid wetting behavior is decided with the component contribution accompanied by the total SE, rather than the single impact previously proposed. Hence, we develop a well-designed parameter, Ψ, to denote versatilely the real OL interfacial property, which is intituled as surface energy component index (SECI, the corresponding unit is $mJ^{-1/2}$ m):

$$\psi = \sqrt{\frac{\varphi_l}{\gamma_l^d + \gamma_l^p}} \tag{14}$$

This parameter further consists of a dispersive part and a polar part:

$$\psi^d = \sqrt{\frac{\varphi_l^d}{\gamma_l^d + \gamma_l^p}} \tag{15}$$

$$\psi^p = \sqrt{\frac{\varphi_l^p}{\gamma_l^d + \gamma_l^p}} \tag{16}$$

The notion presents a well-defined distinction for diversified OLs instead of the SE or dielectric constant (polarity) and set the wetting mechanism apart from counterparts reported to date. As a result, Supplementary Equation 11 can be rewritten to reflect the extent that the inherent liquid SECI and solid SE components affect the theoretical wetting behavior:

$$\cos\theta = 2\sqrt{\gamma_s^d}\psi^d + 2\sqrt{\gamma_s^p}\psi^p - 1 \tag{17}$$

Notably, the intermolecular force of most routine lyophobic membranes is dominantly dispersive part on account of the modification of fluoro-silane or other lyophobic hydrocarbon chains, which has been proved by the previous findings by Pan et al.[6] The known wetting regularity of the above superwetting membranes derives from one extreme case ($\gamma_s^p << \gamma_s^d$) of Supplementary Equation 17 that the polar SE of the membrane can be ignored and the total SE is equal to the contribution of dispersive SE. Therefore, the other extreme case configurated the greater γ_s^p and the smaller γ_s^d may endow the membranes with the opposite wetting trend in theory.

Supplementary Note 3. SE components study based on the flat samples.

It is well-known that the flat silicon wafer is generally used as an ideal substrate to study the wetting behaviors of targeted materials. Supplementary Fig. 17a shows that the silicon wafer coated Capstone FS-50 (as prepared in the Methods) has smooth microtopography, meaning the feasibility of serving as the intrinsic state of FS-SSM. Similarly, the corresponding as-prepared silicon wafer sample can be applied for the situation of PR-SSM, which is strongly smoother than the membrane although the existence of nano-protrusions (Supplementary Fig. 17b). The surface energy and the different components are tested by Fowkes method using two probing liquids with given surface energy components.[20,21] Here we choose water and diiodomethane that are generally recommended in this method to measure the contact angles on the above flat samples. The results of the surface energy and the components of these surfaces are calculated as exhibited in Supplementary Table 2.

Supplementary Note 4. Theoretical computational details.

In order to verify the hypothesis, quantum chemical calculations have been employed using density functional theory to establish whether the positive Ψ^p-driven behaviors (the stronger affinity to higher Ψ^p liquids and vice versa) would be performed in the assumptive situation ($\gamma_s^p >> \gamma_s^d$). Herein, the used Capstone FS-50 molecule is satisfied as the representative molecule to describe the assumed case, of which the quaternary ammonium salt (polar unit) increases the γ_s^p component while the fluorine groups (dispersive unit) diminish the γ_s^d component. The binding energies (ΔE_b) between different OL molecules and Capstone FS-50 molecule are quantitatively calculated to elucidate the corresponding affinity as shown in Fig. 3g. Thereinto the chosen model OLs are composed of polar protic liquids (water, H_2O; formamide, FM; ethylene glycol, EG) and nonpolar liquids (cyclohexane, CYH; dodecane, DOD; dichloromethane, DCM). As shown in Supplementary Table 3, the selected polar protic liquids have higher Ψ^p than the other nonpolar liquids. The important computational result is that the Capstone FS-50 molecule will prefer to be combined with polar protic liquid molecules (higher ΔE_b) rather than nonpolar liquid molecules, indicating a positive Ψ^p-driven tendency in consideration of the Ψ^p values of liquids. Hence, the rationale of the theoretical modeling is expected to render the accessibility to dynamically construct a new-generation membrane system with SECI-tunable wettability (evolved from intermolecular affinity or resistance) for various OLs. The designed capabilities allow us to realize on-demand OL separation, even exhibiting lyophilicity for liquids with higher SE while lyophobicity for liquids with lower SE, which is distinctive from conventional surface tension theory.

Supplementary Note 5. Calculational average pore diameter of the membrane.

In general, the Laplace pressure (ΔP) means the theoretical intrusion pressure (P_{theor}) as shown in Supplementary Equation 18:

$$P_{\text{theor}} = \Delta P = \frac{4\gamma_1 \cos\theta}{D_{\text{pore}}} \tag{18}$$

Where γ_1 is the interfacial tension of the liquid, θ is the contact angle of the liquid on the flat solid surface, D_{pore} represents the average pore diameter of the membrane. For porous materials, the theoretical intrusion pressure can be evaluated by experimental values (P_{exp}) based on the maximum height of liquid supported by the membrane.[22]

$$P_{\text{exp}} = \rho g h_{\text{max}} \tag{19}$$

Where ρ is the density of the liquid, g is the acceleration of gravity, and h_{max} is the maximal height of liquid that can be sustained on the membrane. In theory, we can obtain the average pore diameter of the membrane from the following equations.

$$P_{\text{theor}} = P_{\text{exp}}$$

$$\frac{4\gamma_1 \cos\theta}{D_{\text{pore}}} = \rho g h_{\text{max}}$$

$$D_{\text{pore}} = \frac{4\gamma_1 \cos\theta}{\rho g h_{\text{max}}} \tag{20}$$

Here toluene, cyclohexane, and tetrachloromethane are selected for the measurements. Supplementary Fig. 23a shows the corresponding contact angles on the flat surface and the maximum heights on the membranes. Hence, D_{pore} can be determined according to the above data and the known densities of these liquids in Supplementary Fig. 23b.

Supplementary Note 6. Determination of binary miscible mixture's composition.

Inspired by the SECI-driven surface, we develop a water meter with a set of parallel grooves coated with the resulting TiO_2 nanoparticles, which can detect the volume composition of an unknown miscible OL-water mixture (Supplementary Figs. 33a and b). For instance, acetonitrile (polar aprotic liquid) and water (polar protic liquid) have different wetting behaviors on the paint-coated polytetrafluoroethylene (PTFE) substrate as shown in Supplementary Fig. 33c. Therefore, acetonitrile/water mixtures of varying composition were placed within these channels to exhibit the different wetting lengths, as the water content is varied from 0 to 100 v% with an interval of 10 (Supplementary Fig. 33d). On account of an increase in the polar contribution with the improved water proportion, the wetting length showed a positive trend associated with water composition. According to the standard-fit profile of wetting lengths of mixtures with known compositions, it is convenient to determine the water composition of the arbitrary unknown acetonitrile-water mixture by measuring the corresponding maximum liquid wetting length.

Supplementary Note 7. Evaluation indexes of back extraction and extraction methods

In the proof-of-concept experiment, we adopt three perspectives including back extraction efficiency, volume recovery of back extraction agent, and separation efficiency to evaluate the performance of the in situ back extraction and traditional route. Back extraction efficiency (η_e) refers to the ratio of phenol extraction in the stock solution during the corresponding operation as exhibited in Supplementary Equation 21:

$$\eta_e = \left(1 - \frac{C_t}{C_o}\right) \times 100\% \tag{21}$$

Where C_o and C_t are the phenol concentration at the end of the operation and the original phenol concentration, respectively. Thereinto, the phenol concentration is monitored by ultraviolet-visible (UV-vis) absorption spectrum based on the standard curve of phenol in DCM (Supplementary Fig. 27b). Volume recovery of the back extraction agent (η_v) represents the volume ratio of the back extraction agent that has not been lost after the end of each operation.

$$\eta_v = \left(1 - \frac{V_t}{V_o}\right) \times 100\% \tag{22}$$

Where V_o and V_t are the injected and collected volume of the back extraction agent, respectively. Separation efficiency (η_s) means the degree of resistance to DCM by measuring the residual concentration of DCM in the collected back extraction agent phase.

$$\eta_s = \left(1 - \frac{R_t}{R_o}\right) \times 100\% \tag{23}$$

Where R_o and R_t represent the DCM concentration of the original state and the collected filtrate, respectively. Hence, we can comprehensively assess the capabilities of the two methods through the aforementioned parameters.

Similar evaluation ways are conducted for the extraction operation. It is worth noting that the standard curve of phenol in water should be used to calculate extraction efficiency (Supplementary Fig. 27c).

Supplementary References

1. Wypych, G. *Handbook of Adhesion Promoters*, (Chemtec Publishing Press, Toronto Scarborough, 2018).
2. Cassie, A. B. D. & Baxter, S. Wettability of porous surfaces. *Trans. Faraday Soc.* **40,** 546-551 (1944).
3. Tuteja, A. et al. Designing superoleophobic surfaces. *Science* **318,** 1618-1622 (2007).
4. Tuteja, A., Choi, W., Mabry, J. M., McKinley, G. H. & Cohen, R. E. Robust omniphobic surfaces. *Proc. Natl. Acad. Sci. U.S.A.* **105,** 18200-18205 (2008).
5. Pan, S., Guo, R. & Xu, W. Investigating and biomimicking the surface wetting behaviors of ginkgo leaf. *Soft Matter* **10,** 8800-8803 (2014).
6. Pan, S. et al. Coatings super-repellent to ultralow surface tension liquids. *Nat. Mater.* **17,** 1040 (2018).
7. Lau, K. K. S. Superhydrophobic carbon nanotube forests. *Nano Lett.* **3,** 1701-1705 (2003).
8. Young, T. An essay on the cohesion of fluids. *Phil. Trans. R. Soc. London* **95,** 65-87 (1805).
9. Lafuma, A. & Quere, D. Superhydrophobic states. *Nat. Mater.* **2,** 457-460 (2003).
10. Erbil, H. Y., Demirel, A. L., Avci, Y. & Mert, O. Transformation of a simple plastic into a superhydrophobic surface. *Science* **299,** 1377-1380 (2003).
11. Bird, J. C., Dhiman, R., Kwon, H. M. & Varanasi, K. K. Reducing the contact time of a bouncing drop. *Nature* **503,** 385-388 (2013).
12. Liu, T. & Kim, C. J. Turning a surface superrepellent even to completely wetting liquids. *Science* **346,** 1096-1100 (2014).
13. Fowkes, F. M. Determination of interfacial tensions, contact angles, and dispersion forces in surfaces by assuming additivity of intermolecular interactions in surfaces. *J. Phys. Chem.* **66**, 382-382 (1962).
14. Fowkes, F. M. Attractive forces at interfaces. *Ind. Eng. Chem.* **56**, 40-52 (1964).

15. Owens, D. K. & Wendt, R. C. Estimation of surface free energy of polymers. *J. Appl. Polym. Sci.* **13,** 1741-1747 (1969).

16. Van Oss C. J., Good, R. J. & Chaudhury, M. K. Additive and nonadditive surface tension components and the interpretation of contact angles. *Langmuir* **4,** 884-891 (1988).

17. Van Oss, C. J., Chaudhury, M. K. & Good, R. J. Interfacial Lifshitz-van der Waals and polar interactions in macroscopic systems. *Chem. Rev.* **88,** 927-941 (1988).

18. Graupe, M., Takenaga, M., Koini, T., Colorado, R. & Lee, T. R. Oriented surface dipoles strongly influence interfacial wettabilities. *J. Am. Chem. Soc* **121,** 3222-3223 (1999).

19. Shen, J. et al. Liquid phase exfoliation of two-dimensional materials by directly probing and matching surface tension components. *Nano Lett.* **15,** 5449-5454 (2015).

20. Li, F., Wang, Z., Huang, S., Pan, Y. & Zhao, X. Flexible, durable, and unconditioned superoleophobic/superhydrophilic surfaces for controllable transport and oil-water separation. *Adv. Funct. Mater.* **28,** 1706867 (2018).

21. Wang, Y. et al. Infused-liquid-switchable porous nanofibrous membranes for multiphase liquid separation. *Nat. Commun.* **8**, 575 (2017).

22. Wang, L., Zhao, Y., Tian, Y. & Jiang, L. A general strategy for the separation of immiscible organic liquids by manipulating the surface tensions of nanofibrous membranes. *Angew. Chem. Int. Ed.* **54,** 14732-14737 (2015).
