# Supplementary material for: Universal and tunable liquid–liquid separation by nanoparticle-embedded gating membranes based on a self-defined interfacial parameter
Source: Nat Commun. 2021 Jan 4;12:80. doi: 10.1038/s41467-020-20369-9 (PMC7782719; doi:10.1038/s41467-020-20369-9)
Supplement: Supplementary file 3 — Description of Additional Supplementary Files [file 41467_2020_20369_MOESM3_ESM.pdf]

Description of Additional Supplementary Files

Supplementary Movie 1. The superlyrophobic wetting behavior of toluene on the FS-SSM. When a jet of toluene dyed red flowed to the membrane surface, the spherical droplets left from the surface with low adhesive performance.

Supplementary Movie 2. Toluene drop and water drop bouncing on the surface of FS-SSM. A toluene droplet and a water droplet were released above the FS-SSM that was horizontally placed, respectively. This movie shows that the prepared membrane performs a robust repellency to toluene (nonpolar liquid) and a strong affinity to water (polar protic liquid). The movie was recorded at 10,000 frames per second.

Supplementary Movie 3. The superlyrophobic wetting behavior for water on the PR-SSM. Water jets completely left the surface without wetting or even contaminating the PR-SSM (the water was dyed blue to aid visualization).

Supplementary Movie 4. Toluene and water dropping tests on the PR-SSM. A toluene droplet and a water droplet were dropped above the PR-SSM that was horizontally placed, respectively. This membrane was robust enough to repel an impacting water droplet but wetted by the toluene droplet. The movie was recorded at 10,000 frames per second.

Supplementary Movie 5. Demonstration of gravity-driven separation for immiscible OLs of FS-SSM. In this movie, the mixture of formamide (FM, high Ψ^p liquid, dyed orange) and dodecane (DOD, low Ψ^p liquid, dyed red) can be efficiently separated in accordance with the mode of passing FM but hindering DOD.

Supplementary Movie 6. Reversed immiscible OL separation based on the PR-SSM. For the separation of the mixture of dichloromethane (DCM, high Ψ^d liquid, dyed red) and formamide (FM, low Ψ^d liquid, dyed orange), DCM selectively permeated through the PR-SSM while FM was retained, which worked in the reverse separation mode from FS-SSM.

Supplementary Movie 7. Self-adaptive OL gating in a designed fluidic separation device based on FS-SSM and PR-SSM. Utilizing the self-adaptive two outlets fluidic gating device integrating FS-SSM and PR-SSM, the FM/DOD mixture can be automatically and conveniently separated into two phases. FS-SSM served as the FM outlet and PR-SSM worked as the DOD outlet, respectively.

Supplementary Movie 8. Continuous in situ back extraction of FS-SSM. The whole process was carried out by fixing the vertically placed FS-SSM with the designed real-time extraction device. Phenol initially enriched in DCM solution can be concentrated into NaOH aqueous solution (back extraction agent). After a short mixing, the back extraction agent containing phenol can flow out through the membrane owing to its affinity with water and repellence against DCM.

Supplementary Movie 9. Traditional back extraction by using the separating funnel. This movie displays the traditional back extraction of phenol in DCM solution with the assistance of NaOH aqueous solution, which requires multiple procedures comprising drastic mix, gravity settlement, and static separation.

Supplementary Movie 10. Continuous in situ extraction of PR-SSM. In this movie, fresh DCM (extraction agent) was constantly kept dropping into the device filled with an aqueous phenol solution, and a continuous extracting process was available by selectively and automatically passing the as-extracted liquid through the PR-SSM.

Supplementary Movie 11. Traditional extraction by using the separating funnel. The traditional extraction of phenol in aqueous solution was performed with the extracting liquid (DCM), in which complicated procedures of mixing, transferring, centrifuging, and waiting for phase equilibrium were equipped.
